# Nanoparticles as drug delivery systems in the treatment of oral squamous cell carcinoma: current status and recent progression

**DOI:** 10.3389/fphar.2023.1176422

**Published:** 2023-05-24

**Authors:** Shuxia Cui, Hanzhe Liu, Guanglin Cui

**Affiliations:** ^1^ Henan Stomatological Hospital, The First Affiliated Hospital of Zhengzhou University, Zhengzhou, China; ^2^ School of Stomatology, Wuhan University, Wuhan, China; ^3^ Faculty of Health Science, Campus Levanger, Nord University, Levanger, Norway

**Keywords:** nanoparticles, drug delivery system, oral squamous cell carcinoma, anticancer drug, treatment

## Abstract

Oral squamous cell carcinoma (OSCC) is a common human malignancy with an estimated incidence of around 377,713 new cases worldwide in 2020. Despite the advance in clinical management, some of OSCC patients still miss the opportunity of completable resection of tumor, and have to accept medical therapies, e.g., chemotherapy, radiotherapy, or immunotherapy when the disease develops into the advanced stage. However, these therapies have been reported to be far from ideal due to the low efficiency of conventional delivery approaches. To obtain a better therapeutic effect, considerable attempts have been made toward to develop an effective drug delivery system (DDS). Nanoparticles (NPs) including inorganic NPs, polymer NPs, lipid NP, extracellular vesicles and cell membrane-based NPs have been evaluated as the better DDS candidates that can specifically accumulate in the tumor microenvironment along with a large amount of blood vessels. Emerging evidence suggested that NPs formulated with anticancer drugs including chemotherapeutic drugs, radiotherapy and immunotarget antibodies could remarkably improve the release and increase concentration of these drugs at the tumor site and show a better therapeutic efficacy, suggesting that NPs might serve as promising DDSs in the treatment of OSCC. Therefore, we have conducted this review to summarize recent progression and current status of diverse NPs as DDSs in this research field.

## 1 Introduction

Oral squamous cell carcinoma (OSCC) is the most common type of oral cancers, with an approximately 70% increase in incidence over the past 20 years (Gulland, 2016; Bugshan and Farooq, 2020). The estimated annual incidence of OSCC in 2020 was around 377,713 new cases worldwide, with Asia having the highest number of cases, followed by Europe and North America, according to statistical data obtained from 185 countries (Sung et al., 2021). The usual treatment for patients with early stage OSCC is surgery that can totally remove the tumors. The main clinical management for patients with advanced stage OCSS is diverse medical therapies i.e., radiotherapy, chemotherapy, immunotherapy, and combination therapy (Nandini et al., 2020; Deshmukh et al., 2021). Although these medical therapies have been optimized over the last several decades, however, they are still far from ideal therapeutic efficacy (Siegel et al., 2020; Surer et al., 2021) and the overall 5-year survival rate is less than 60% (Yu et al., 2019). Studies indicate that one of the main mechanisms responsible for such low therapeutic effect is the low efficacy of drug delivery system (DDS) that results in anticancer drugs being too low at the tumor site to exert their anticancer effects (Zhang et al., 2020a). Therefore, considerable attempts in the development of novel DDSs that can notably increase the concentration of drugs at the tumor site have been made (Ketabat et al., 2019). Recent studies have demonstrated that nanoparticles (NPs) as DDSs might reduce the toxicity and improve the safety and specificity of drugs, and represent as a promising tool for the treatment of OSCC (Calixto et al., 2014; Sah et al., 2018; Ketabat et al., 2019).

Theoretically, NPs usually refer to particles with a particle size between 1 and 100 nm. Extensive studies show that NPs can be specifically absorbed into the interstitial space of the tumor and are not rapidly cleared through enhanced permeability and retention (EPR) effects, that is a really stablished and important process and can induce in an enhanced accumulation of formulated anticancer drugs within the tumor microenvironment and result in a improve therapeutic effect (Nakamura et al., 2016). Currently, considerable NPs has been developed and evaluated, results showed an encouraged result in the treatment of OSCC.

CC. We have, therefore, summarized recent advances in the applications of major NPs as novel DDSs in the treatment of OSCC. These findings might help researchers and clinicians to better understand the effects of NPs in combining with anticancer medical therapies and to design novel therapies that significantly improve therapeutic response in patients with OSCC.

## 2 Different NPs as DDSs in enhancing the efficacy of anticancer drugs in OCSS

Modern nanotechnology is involved in various fields of clinical research and science, where such NPs-based DDSs are of major interest (Calixto et al., 2014). Several NPs have been developed as DDSs in the treatment of OSCC, which included inorganic NPs, extracellular vesicles, polymer NPs, and lipid NPs, cell membrane-based NPs and nanoenzymes (Calixto et al., 2014; Chen et al., 2023a; Mabrouk et al., 2023; Ortega et al., 2023). The improved therapeutic effect of NPs combined with anticancer agents has been demonstrated (De Felice et al., 2019). For example, Mukherjee et al. (2022) treated OSCC cell line (KB 3-1 cell) for 24 h with cetuximab (a epithelial growth factor receptor (EGFR) inhibitor) and NC in various doses. Comparing NC to cetuximab, they found a concentration-dependent cancer cell death that was remarkable. Therefore, NPs as promising DDS candidates have shown a great potential to increase cytotoxicity and overcome resistance to anticancer therapies, in the treatment of OSCC.

The following paragraphs will discuss the potential effect of NPs as effective DDSs individually.

### 2.1 Inorganic NPs

Inorganic NPs have been broadly used as DDSs in the treatment of tumors due to their unique physicochemical properties, e.g., facile preparation, excellent biocompatibility and wide surface conjugation chemistry (Wang et al., 2016), including various gold NPs, platinum NPs, iron NPs, manganese NPs, and silicon NPs, etc. For example, gold NPs have unique optical and surface plasmon resonance properties, making them particularly suitable for the ultra-sensitive detection and imaging-based therapeutic techniques needed to treat cancer (Singh et al., 2018). Due to the strong anticancer effect of platinum, platinum NPs also possess efficient anticancer ability despite behaving differently from platinum-containing compounds (Abed et al., 2022). Another commonly used inorganic NPs, iron oxide, is superparamagnetic at certain sizes and has been widely used as a contrast agent and drug delivery vehicle to treat cancer (Arias et al., 2018). A reduced toxicity, greater tolerance to organic solvents, and higher bioavailability than free drugs have been demonstrated in experiments (Ketabat et al., 2019). The characteristics of each inorganic nanoparticle are summarized in Table 1.

**TABLE 1:** Exhibitions of inorganic NPs as drug delivery system in OSCC.

Inorganic nanoparticles	Experimental models/cells	Effects	References
GNP	NIH-3T3、UPCI-SCC-131	Enhance efficacy of radiotherapy	Surer et al. (2021)
GNP-CDDP	NIH-3T3	Reduce OSCC cell activity	
	UPCI-SCC-131	Enhance efficacy of radiotherapy	
GNP-CDDP-CTX	NIH-3T3、UPCI-SCC-131	Reduce OSCC cell activity	
5-Fu、CPT、FGFR1i combined with AuNSs	Chemical induced hamster buccal pouch carcinoma model (HBPC)	FGFR1i-AuNSs Induce a more pronounced sub-G1 cell population	Abdel Hamid et al. (2021)
		FGFR1i-AuNSs induced higher tumor reduction rates than other groups	
		AuNS selectively enhance the therapeutic effect of small molecule inhibitors	
GA-AuNPs	Cal-27	GA-AuNPs induce cytotoxicity	Gamal-Eldeen et al. (2021)
		Inhibition of cellular hypoxia effects in a dose-dependent manner	
		Induces early and late apoptosis in CAL-27 cells	
		Significantly decreased the expression of miR-210 and miR-21	
		Inhibits HIF-1α and c-Myc	
		Potential to reduce hypoxic and hypoxic expression levels	
GNP、PD-L1、NTP	SCC-25	Enhance cellular intake of drugs	Liu et al. (2020)
		Reduce OSCC cell activity	
		Promote apoptotic protein expression	
(PDPN Ab)-AuNP-DOX	Noncancerous 293T cells	Reduced cytotoxicity	Liu et al. (2020)
	Cal-27	Enhance cellular intake of drugs	
		Enhance anticancer efficacy with PTT	
	Xenograft Model by Subcutaneous Injection of CAL-27	Enhance anticancer efficacy of PTT, increased DOX concentration in tumor site	
	HSC-3 、HaCat	Enhanced apoptosis in tumor cells	Essawy et al. (2021)
Ph sensitive DOX-s-AuNPs	Chemical induced hamster buccal pouch carcinoma model (HBPC)	DOX-N-N-AuNPs-treated animals had significantly reduced tumor size and high survival rates without hematopoietic adverse effects	
Ph stable DOX-N-N-AuNPs		DOX-N-N-AuNPs enhance apoptosis in tumor cells	
GNSb	Mouse L929 fibroblast cell line, CAL-27	Selective toxicity higher cellular uptake of drugs	Chen et al. (2021)
		Combined radiation enhances cytotoxicity, forms more ROS, induces more pronounced DNA double-strand breaks and arrest in G2/M phase	
	OSCC Xenograft Model Established by Injecting CAL-27 into Mice	Combining radiation significantly inhibits tumor growth	
	OSC-19	CRDs induce higher cytotoxicity	Zuo et al. (2020)
		CRDs are highly biocompatible	
Cu_2-x_-S-RB@DMSN-AE105 (CRDA)	Xenograft Model by Subcutaneous Injection of OSC-19 cells in Mice	CRDs accumulate faster and higher at tumor sites inhibit tumor cell proliferation	
Cu_2-x_-S-RB@DMSN Nanoparticles (CRDs)		Reduce tumor cells and increase the proportion of apoptotic areas	
		Combined PTT and SDT eradicated xenografts and prolonged mouse survival	
SPION	OSCC cells	Decreased SDH activity, increased ROS levels, MMP collapse, mitochondrial swelling, and cytochrome c release in mitochondria of OSCC cells	Afrasiabi et al. (2021)
		Decreased cell viability, increased LPO levels and caspase-3 activation in OSCC cells	
PtNCP (platinum nanocomposite beads)	HSC-3-M3	Inhibit OSCC cell activity in a dose-response manner	Tanaka et al. (2019)
		Promote cytotoxicity via LDH	
	Xenograft Model by Subcutaneous Injection of HSC-3-M3 in Mice	Suppress tumor growth	
		Induce tumor cell necrosis	
Nano-SHAP contained cisplatin and carboplatin	HSC-4、KOSC、SAS	Suppress tumor cell proliferation	Murata et al. (2018)
UCA-cMBP	Cal-27、A549、HeLa 、4T1 、MCF-10 A	Better targeting ability to Cal27 cells	Lin et al. (2021)
		Enhanced cellular uptake of drugs	
		Inhibit the invasion and metastasis of Cal27 cells	
		Concentration-dependent apoptosis in tumor cells	
	Mice were injected intravenously with UCA-cMBP	Good clearance from the body without significant organ damage	
Anti-GFR-PEG-TiO 2-UCNs	CAL-27、H596 (HTB-178) 和 H460 (HTB-177)、A549 (CCL-185)、MCF-7 (HTB-22)、Hep-G2 (HB-8065) 和 NHF (IMR-90)	Faster and more efficient internalization by OSCC cells	Lucky et al. (2016)
		Selectively kills EGFR expressing cells	
	Xenograft Model Established by Injecting OSCC cells into Mice	Mice exhibited significant tumor growth delay and higher survival	
S-CD	UM1 cell line derived from HNSCC patient	The ability to generate singlet oxygen under light is higher than that of traditional 5-ALA	Li et al. (2020a)
		When it is taken up by cells under light, the cell viability decreases immediately, and it is concentration-dependent	
		Exhibits low cytotoxicity in the absence of light	
NCQD-HCS	FaDu cell, HaCaT cell	NCQD-HCS was internalized by cells and induced a significant thermal ablation effect in FaDu cells when exposed to 980 nm near-infrared laser light	Das et al. (2019)
GQD-PEG	SCC-25、SCC-9 、HOK	Has strong phototoxicity	Zhang et al. (2020b)
		Low cytotoxicity, satisfactory solution stability and excellent endocytosis	
	Xenograft Model by Subcutaneous Injection of SCC VII in Mice	Significantly increases host immune-associated CD8^+^ T cells and pro-inflammatory cytokines	
		Robust ablation of OSCC and tumor-targeted accumulation under photoirradiation	
H-MnO 2-PEG/TP nanoshells	CAL-27、SCC-7	Cell proliferation, colony formation, and migration abilities were significantly reduced	Zhang et al. (2020b)
		Enhanced apoptosis in tumor cells	
		Hypoxia-inducible factor-1α (HIF-1α) was downregulated	
	Xenograft Model by Intravenous or Subcutaneous Injection of CAL-27 in Mice	The uptake ratio of drugs between tumor and normal organs in mice was significantly increased	
		Most tumor cells are severely damaged	
		Enhanced apoptosis	

#### 2.1.1 Gold NPs (AuNPs or GNPs)

Gold NPs, as DDS, have been extensively studied, and promising results were observed. For example, Abdel Hamid et al. (2021) used AuNS as a drug delivery vectors to evaluate the combination effect of cytotoxic chemo-drugs 5- fluorouracil (5-Fu), camptothecin (CPT), and small molecule inhibitor fibroblast growth factor receptor1-inhibitor (FGFR1i) both *in vitro* and *in vivo* in the oral cancer, survival analysis data showed that Syrian golden male hamsters treated with conjugating AuNSs with FGFR1i, 5Fu, and CPT could remarkedly enhance survival time to more than 27 days, which was much longer than animals treated with free FGFR1i (survival time 13.5 days). In addition, animals treated with FGFR1i-AuNSs induced the highest tumor volume reduction with a 2-fold decrease (−63.09%) compared with those injected by CPT-AuNSs (−32.1%) or 5Fu-AuNSs (−43.4%) (Abdel Hamid et al., 2021). When Gamal-Eldeen et al. (2021) examined the effect of gum arabic encapsulated gold NPsNPs (GA-AuNPs) on the hypoxia regulators in tongue squamous cell carcinoma (CAL-27 cells) *in vitro*, they found that GA-AuNPs can remarkably reduce cell viability with IC50 of 392.3 and 247.3 μg/mL after 24 and 48 h, respectively, and increase CAL-27 cell death rate via enhanced apoptosis. In addition, GA-AuNPs significantly inhibited hypoxia production in a dose-dependent manner, which could be via the decreased expression of hypoxia-regulating miRNAs (miR-210 and miR-21), hypoxia inducible factor-1 *α* (HIF-1α) and c-Myc (factors influence cell survival and angiogenesis) by GA-AuNPs (30% IC50, for 48 h) (Gamal-Eldeen et al., 2021). Park et al. (2021) investigated the effectiveness of a novel combination therapy by using gold nanoparticles (GNP) conjugated to anti programmed cell death protein ligand 1 (PD-L1) antibodies and nonthermal plasma (NTP) in PD-L1 expressing SCC-25 cells. They found that immunotarget anti- PD-L1 antibody and NTP-conjugated gold NPs could specifically bind to SCC-25 cells and induced an increase in the selective uptake of anti-PD-L1 antibody + GNP on SCC-25 cells, but significantly reduced tumor cell viability. In addition, the expression of apoptosis-related proteins and the number of dead cells were remarkably increased after treatment with anti-PD-L1 antibody + GNP + NTP. Thus, their results suggested a synergistic therapeutic effect than monotherapy.

In addition, some gold NPs can also increase the respond to environmental conditions such as light and heat. These features allowed gold NPs conjugated with radiotherapy, phototherapy, or photothermal therapy to potent their therapeutic effect. Indeed, Inanc Surer et al. (2021) used nanodrug complex containing cetuximab (CTX) and cisplatin (CDDP) conjugated with gold NPs to evaluate its therapeutic effects in both radioresistant oral cancer cell line (UPCI-SCC-131) and fibroblast cell line (NIH-3T3) *in vitro*. The results showed that the combination of nanodrugs GNP-CTX or CDDP with radiotherapy significantly result in 2-fold and 9-fold greater decrease in the colony number of radioresistant oral cancer cells than radiotherapy alone or free CTX combined with radiotherapy respectively (Surer et al., 2021). Furthermore, PEG-stabilized, PDPN antibody and doxorubicin (DOX)-conjugated gold NPs and Ph-stabilized DOX-AuNPs (DOX-NN-AuNPs) in combining with laser treatment could significantly increase apoptosis and decrease the growth of tumor cells (Liu et al., 2020; Essawy et al., 2021). The study by Chen et al. (2021) showed that gold nano-sesame beads (GNSbs) in combining with 2 Gy irradiation significantly increased the cytotoxic activity and decreased the proliferation activity of CAL-27 cells in a concentration-dependent manner, and the formation of reactive oxygen species (ROS) as compared with irradiation alone (Chen et al., 2021). In mice with CAL-27 orthotopically injected into the oral wall model, radiotherapy combining with GNSbs (delivered every 3 days for a total dose of 10 Gy (irradiation × 5 times) and 54 mg kg−1 GNSbs) treatment resulted in a significantly decrease in average tumor size from day 0 to day 21 (Chen et al., 2021). These results suggest that GNSbs is a promising radioenhancer that enhances the therapeutic effect of radiotherapy in OSCC.

#### 2.1.2 Mesoporous silica NPs (MSNs)

Due to their distinguishing characteristics, such as pore volume, large specific surface area, controllable particle size, and great biocompatibility, MSNs as DDSs have attracted considerable attention and been intensively studied (Wang et al., 2015). Shi et al. (2019) reported that delivery of MTH1 inhibitor (TH287) and MDR1 siRNA via hyaluronic acid-based MSN in CAL-27 OSCC cells could induce more effective anticancer effects *in vitro*. In male Balb/c mice with subcutaneously injecting 2 × 106 CAL27 cells model, both SiTMSN and HA-siTMSN showed an antitumor effect. However, HA-siTMSN induced 4-fold decrease in the tumor volume compared to that of control and 2-fold compared to that of the siTMSN. indicating its stronger anti-oral cancer efficacy. By using the urokinase plasminogen activator receptor (uPAR)-targeting ligand AE105 decorated dendritic mesoporous silica nanoparticles (DMSN) encapsulating photonic active ultrasmall Cu2−xS NPs and sonosensitizer Rose Bengal (RB) (Cu2−xS-RB@DMSN-AE105, abbreviated as CRDA), Zuo et al. (2020) showed an increase of CRAD at tumor site and induced higher cytotoxicity, inhibit cell proliferation, reduce tumor cells by increasing apoptosis rate in OSCC cells. Furthermore, they further evaluated synergetic therapeutics of CRDAs against OSCC xenografts in BALB/c mice, their results showed that CRAD combined with photonic hyperthermal therapy (PTT) or sonodynamic therapy (SDT) in mice elicited enhanced therapeutic efficacy with an inhibition rate of 103.4%. The eradication of the xenografts in the CRDAs + US + laser group were enhanced, the survival rate was increased and living time of mice was prolonged (Zuo et al., 2020).

#### 2.1.3 Other inorganic NPs

There are other inorganic NPs have been assessed. For example, both platinum nanocomposite (PtNCP) beads and superparamagnetic iron oxide NPs (SPION) were found to be cytotoxic to OSCC cells (Tanaka et al., 2019; Afrasiabi et al., 2021), Tanaka et al. (2019) reported that PtNCP could significantly inhibit tumor growth and caused more pathological necrosis area in subcutaneously xenografting mice with human squamous cell carcinoma cells, HSC-3-M3. They found that the mean tumor volumes in HSC-3-M3 cells xenografts in mice receiving PtNCP treatment for 14 days were significantly reduced than that in untreated group (PtNCP treated group vs. untreated group: 91.38 vs. 206.18 mm^3^). The inhibition rate on tumor volume in PtNCP treated group was 44.32% compared to untreated group (Tanaka et al., 2019). Histologically, necrotic areas in tumors were frequently observed in the PtNCP beads-treated group compared to the untreated group (Tanaka et al., 2019). Their findings suggest an inhibitory effect of PtNCP on OSCC.

The use of hollow mesoporous MnO_2_ (H-MnO_2_) nanoshells formulated with docetaxel and cisplatin and highly dispersed calcined hydroxyapatite NPs (nano-SHAP) loaded with zoledronic acid (ZA) were both shown to inhibit OSCC cell proliferation (Murata et al., 2018; Zhou et al., 2021). Murata et al. (Zhou et al., 2021) evaluated the effect of hydroxyapatite anoparticle as a new DDS on OSCC cell line, their results showed that Nano-SHAP with ZA suppressed remarkably OSCC tumor cell growth, damage tumor cells and upregulate apoptosis in OSCC *in vivo*.

By using dual-modal optical imaging rare earth nanoparticle (RENP) probes with peptide functionalization (RENP@C@Au (UCA)), Lin et al. (2021) demonstrated that it could effectively target Cal-27 tongue squamous cell carcinoma (TSCC) cells, enhance cell uptake of drug, inhibit invasion and metastasis, cause concentration-dependent apoptosis, and has an excellent *in vivo* clearance rate without causing significant organ damage.

In addition, photothermal Therapy has been shown to be a potential therapeutic approach in treating OSCC (Ran et al., 2023). Based on the properties of near-infrared (NIR) excitable upconversion NPs (UCN), photosensitizers such as photodynamic therapy (PDT) agents (Lucky et al., 2016), sulfur-doped carbon dots (S-CD) (Li et al., 2020a), nitrogen-rich mesoporous carbon Nanosphere void spaces trapping ultra-small nitrogen-doped carbon quantum dots (NCQDs) (Das et al., 2019) have been reported to show an enhanced therapeutic effect. Graphene quantum dots (GQDs) as the photosensitizer and GQD-polyethylene glycol (PEG) obtained by combining with PEG (Zhang et al., 2020b) have also been found to enhance the antitumor effect in the combination with different degrees of light in subcutaneously xenografting mice with SCC VII cells. They found that SCC VII tumor-bearing C3H mice treated with GQD-PEG plus irradiation induced tumor size reduction exceeding 70%, compared with control groups, they concluded that such antitumor effect might be attributable to the efficient tumor accumulations mediated by the EPR effect and the high ^1^O_2_ toxicity induced from photoactivity (Zhang et al., 2020b).

For the convenience of readers, the characteristics of these NPs are summarized in Table 1.

### 2.2 Extracellular vesicles (EVs)

EVs are natural nano-sized lipid bilayer vesicles released by all cell types and can be found in biological fluids such as blood, saliva, breast milk, cerebrospinal fluids and malignant ascites (Elsharkasy et al., 2020). Multiple studies have shown that EVs have a similar specific cellular tropism, which function as target vesicles for specific tissues and/or organs. Exosomes (EXO), nanoscale vesicles secreted by various cells, have the ability to cross biological barriers, including the most impenetrable blood-brain barrier (Batrakova and Kim, 2015; Wiklander et al., 2015) and attract a high attention (Wiklander et al., 2015).

Qiu et al. (2020) found that the loading and release of CTX from mesenchymal stromal cell (MSC)-derived EXO (MSC-EXO) significantly inhibited tumor growth by the activation of PI3K, Akt and mTOR and the induction of apoptosis in SCC-25 cells in a dose-dependent manner. They further showed that such inhibitory degree was in an effective synergistic and pharmacological effect. By developing a pH/light sensitive drug system based on milk-EXO for OSCC therapy, Zhang et al. (2020c found that a bovine milk EXO-based EXO-doxorubicin (DOX)-anthracene endoperoxide derivative (Exo@Dox-EPT1) could remarkably increase cellular uptake, release of DOX under an acidic microenvironment and generate ROS in combination with 808 nm NIR laser stimulation [31]. This new milk-EXO-based DDS exhibited an significant control effect on drug-release, biocompatibility and OSCC cell growth.

Apart from its inhibitory effect on OSCC cells, bitter melon-derived extracellular vesicles (BMEV) have been found to suppress the expression of NLRP3 and IL-1β and reduce the tumor resistance to 5-FU via an increase in apoptosis rate in OSCC cells (Yang et al., 2021). By using γδ T cell-derived extracellular vesicles (γδ TDE) loaded with miR-138, Li et al. (2019) showed that γδ TDE could directly inhibit OSCC tumor progression by up-regulating anti-tumor immunity through enhanced CD8 + T cells in pre-immunized immunocompetent C3H mice.

Regarding the role of EVs as DDSs in treating OSCC, we have summarized it in Table 2.

**TABLE 2:** Exhibitions of extracellular vesicles (EVs) as natural nano drug delivery system in OSCC.

EVS	Models/cells	Effects	References
BMEV	CAL-27、WSU-HN6	Induces S-phase arrest to inhibit cell proliferation in a dose-dependent manner	Yang et al. (2021)
		Upregulation of caspase 3 and stimulation of ROS generation to induce apoptosis in OSCC cells	
		Downregulation of NLRP3 expression	
BMEV、5-FU	OSCC xenograft model by injecting CAL-27 into mice	Decreased expression of NLRP3 and IL-1β	
		Reduce tumor resistance to 5-FU	
		Increased apoptosis rate of OSCC cells	
MSCT-EXO/CTX	SCC-25	Inhibits the activation of PI3K, Akt and mTOR	Qiu et al. (2020)
		Induces apoptosis of SCC25 tumor cells in a dose-dependent manner	
	OSCC xenograft model by injecting SCC-25 into mice	Tumor size shrinks	
		Increased tumor suppression rate	
Exo@Dox-EPT1	HSC-3、SCC-9、CAL-27	Increase cellular uptake	Zhang et al. (2020c)
		Sustained release of Dox under acidic conditions	
		Efficient generation of ROS after stimulation with 808 nm NIR laser	
		The cytotoxicity of NP 808 group to cancer cells was significantly higher than that of free Dox group	
	Mouse Models of HSC-3, SCC-9, and CAL-27 Xenograft Tumors	The tumor growth in the NP 808 group was effectively inhibited and almost disappeared after treatment	
		Enhance drug accumulation and retention in tumor tissue	
		Has good biocompatibility	
γδ TDE loaded with miR-138	Cal-27 、SCC-VII	Regulation of antitumor immunity by CD8^+^ T cells	Li et al. (2019)
	Xenograft Models Established by Subcutaneous Injection of Tumor Cells in Immunodeficient Nude and Immunocompetent C3H Mice	Preimmunized immunocompetent C3H mice and directly inhibited OSCC tumor growth	

### 2.3 Polymer NPs

Polymer NPs, including natural polymers and semi-synthetic polymer NPs, possess the promoting potential to cellular permeability (Charbe et al., 2020; Xia et al., 2021). Among them, some polymer NPs with good biocompatibility and controlled drug release as targeted DDSs have been evaluated (Rizvi and Saleh, 2018).

Kurakula and Naveen (2020) loaded quercetin-doped chitosan-coated simvastatin (SIM) NPs in an *in situ* gel (ISG) (SIM-QRC NP-loaded ISGs) and demonstrated an delay of drug release, which resulted in a markedly increase in caspase-3-mediated apoptosis and tumor suppressor protein expression in tongue SSC cells. Enășescu et al. (2021) found that lutein and poly (d,l-lactide-co-glycolide) (PLGA) NPs potentially downregulated matrix metallopeptidase 9 level in human OSCC cells, which has been recognized as a protecting factor that could against local invasion in tumors (Chakraborty et al., 2023). Furthermore, both DOX-loaded catechol (Cat)-modified chitosan/hyaluronic acid (HA) NPs (Cat-NPs) and phloretin-loaded chitosan NPs (PhCsNPs) could increase the release of DOX, which significantly inhibited cancer cell growth and increased apoptosis rate in human oral cancer cells (Mariadoss et al., 2019; Pornpitchanarong et al., 2020). Endo et al. (2013) reported that commonly used chemotherapy drug cisplatin-carrying polymer micelles (NC-6004) showed an equivalent antitumor effect as free cisplatin *in vivo*, although the inhibitory effect on the growth of oral cancer cells was less than that of free cisplatin *in vitro*. The authors also found that the toxicity of NC-6004 to kidney and the incidence of lymphatic metastasis were lower than free cisplatin (Endo et al., 2013). Li et al. (2020b) found that polylactic acid (PLA) combined with CDDP-chloroquine (CQ) NPs (CDDP/CQ-PLA NPs) could induce a stronger activation of caspase-3 pathway and the induction of ROS than PLA combined with CDDP NPs (CDDP-PLA NPs), which leaded to an increased rate of caspase-dependent apoptosis and but lower rate of autophagy in OSCC (Li et al., 2020b).

Polymer NPs with targeting function have broad prospects as DDS. All-trans retinoic acid (ATRA) can be loaded onto PLGA-PEG and then modified with PD-L1 antibody to prepare ATRA- Targeted *α*-t- FU-PLGA NPs (Srivastava et al., 2019), glutathione (GSH)-sensitive and folic acid (FA)-targeted paclitaxel-loaded NPs (FA-PEG-SS-PCL@PTX, FA-NPs) (Fan et al., 2020a), cRGD targeted polycarbonate (PCA) copolymer-based NPs (NanoPCA) loaded with DOX (NanoPCA-cRGD) (Yunxia et al., 2018) have been proved to have a good targeting effect, which can precisely release and enhance antitumor efficacy of diverse anti-tumor drugs. To evaluate the potential effect of polymer NPs on immune checkpoint inhibitor in oral dysplasia and squamous carcinoma cells, Chen et al. (2020) examined CD8^+^ T cells surrounding PD-L1-positive cells in the tumor microenvironment. Both *in vitro* and *in vivo* results showed that CD8^+^ T cells were more activated after ATRA-PLGA-PEG-PD-L1 treatment (Chen et al., 2020).

Finally, polymer NPs combined with radiotherapy also exhibited an improved therapeutic effect on OSSC. Lang et al. (2020) reported that radiation combined with capivasertib-encapsulated cathepsin B (CTSB)-reactive NPs could significantly reduce tumor cell viability and increase the rate of apoptosis than radiation alone, or free capivasertib in OSCC cells, providing a novel strategy to improve therapeutic strategy for patients with radiation resistant.

The effects of polymer NPs as DDSs in the oral cancer were summarized in Tables 3, 4.

**TABLE 3:** Exhibitions of polymer NPs as drug delivery systemin in OSCC.

Polymer nanoparticles	Models/cells	Exhibitions	References
ATRA-PLGA-PEG-PD-L1	DOK、CAL-27	fast cellular uptake of drugs	Chen et al. (2020)
		Significantly inhibit cell proliferation induce apoptosis	
	Xenograft model established by subcutaneous injection of SCC-7 cells in mice	Specifically targeting tumor cells	
		Enhance anticancer activity	
		Reduce side effects of drugs	
		Activate CD8^+^ T cells and PD-L1-positive cells in the tumor microenvironment	
CDDP/CQ-PLA NPs and CDDP-PLA NPs	CAL-27 cells	Reliable performance in nano drug loading and drug release	Li et al. (2020b)
		CDDP/CQ-PLA NPs lead to more caspase-dependent apoptosis than CDDP-PLA NPs through the caspase-3 pathway and induce more ROS production	
Cathepsin B-reactive nanoparticles encapsulate capivasertib (Nano-cap)	SCC-35、CAL-27、HN6、HN12 cells	Reduce tumor cell viability	Lang et al. (2020)
		Induce tumor cell apoptosis	
	Xenograft model established by injecting HN12 cells into NSG mice	Induces apoptosis of OSCC tumor cells	
		Shrink the tumor	
α-t-FU-PLGA NPs	SCC-15 cells	Higher cytotoxicity	Srivastava et al. (2019)
		Higher cell penetration	
		Higher accumulation of cancer cells	
FA-PEG-SS-PCL@PTX, FA-NPs	HSC-3 cells	PTX in FA-NPs can be precisely released and enhance cell growth inhibition in FA-overexpressing HSC3 cells	Fan et al. (2020a)
	Xenograft model established by subcutaneous injection of HSC-3 cells in mice	FA-NPs can accumulate in HSC3 cells and exhibit greater antitumor efficacy than free PTX and PEG-SS-PCL@PTX treatment with reduced side effects	
ISG loaded with SIM-QRC NP	HSC-3 cells	The drug is released slowly and takes 96 h to reach the plateau	Kurakula and Naveen (2020)
		Significantly increased apoptosis mediated by caspase-3	
		Increased tumor suppressor protein levels	
Lut Nps	BICR10 OSCC cells (ECACC 04072103)	Downregulates MMP-9 levels	Enășescu et al. (2021)
NanoPCA, including P(CA-co-LA), P(CA-co-LA)-g-MPEG, P(CA-co-LA)-g-PEG-cRGD]		Excellent drug loading capacity (9.1% mass ratio) long-term stability in water	Yunxia et al. (2018)
	SCC15 cells	The cytotoxicity and apoptosis induction of DOX released by NanoPCA are not as good as that of free DOX	
		Slow and stable release, faster release under acidic conditions	
		NanoPCA-cRGD can enhance DOX uptake by SCC-15 cells through active targeting of cRGD	
	Xenograft model established by subcutaneous injection of SCC15 cells in mice	DOX-loaded NanoPCA significantly inhibits tumor growth and prolongs the survival time of mice	
		No obvious adverse reactions	
Cat-NPs loaded with DOX	Pig buccal oral mucosa	Excellent mucoadhesive ability, high drug loading and slow drug release	Pornpitchanarong et al. (2020)
	HN22 cells	More extensive uptake and accumulation in cancer cells	
PhCsNPs	HEK-293、KB cells	PhCsNPs release phloretin in the acidic environment of cancer cells with sustained and controlled drug release	Mariadoss et al. (2019)
		Enhances mitochondria-mediated apoptosis by inducing intracellular ROS production, stimulating oxidative stress, depleting cellular antioxidants, and cell cycle arrest	
siTMSN 和 HA-siTMSN	CAL-27 cells	Effective control of drug release and internalization in cancer cells	Shi et al. (2019)
		The combination of TH287 + MDR1 siRNA is more effective in inducing anticancer effects	
	Xenograft model established by subcutaneous injection of CAL-27 cells in mice	SiTMSN and HA-siTMSN significantly reduce tumor size	
NC‐6004	OSC-19、OSC-20、HSC-3 、HSC-4	NC‐6004 was significantly less growth inhibitory than free CDDP	Endo et al. (2013)
	Xenograft model established by subcutaneous injection of OSC-19 cells in mice	NC‐6004 and free CDDP show equivalent antitumor effects	
		NC-6004 is less toxic to the kidney than free CDDP	
		NC-6004 also has a lower incidence of lymphatic metastasis	

**TABLE 4:** Exhibitions of lipid NPs as drug delivery systemin in OSCC.

Lipid nanoparticles	Cells/models	Exhibitions	References
SLN loaded with PTX, 5-FU and AA individually	4-NQO-induced OSCC mouse model	Combination of PTX-loaded SLN and AA-loaded SLN has greater efficacy in OSCC	Bharadwaj et al. (2019)
	SAS cells	LPC shows greater cancer cell killing than CDDP	Gusti-Ngurah-Putu et al. (2019)
	SCC model established by subcutaneous inoculation of SAS cells in mice	PDT + LPC significantly reduced tumor volume by approximately 112%, stronger than LPC, PDT + CDDP or CDDP groups	
LPC NPs		Reduced tumor growth rate	
LPC NPs		Significantly less side effects	
		PDT + LPC or LPC treatment had minimal adverse effects on kidney injury compared with CDDP or PDT + CDDP group	
		Strong additive effects of PDT enhance the chemotherapeutic efficacy of LPC NPs	

### 2.4 Lipid NPs

Lipid NPs are delivered into tumor tissue through the passively targeted EPR effects. To obtain an selective targeting efficacy, ligands can also be attached to the surface of lipid NPs. Lipid NPs are ideal carriers for drugs with low water solubility because of their ability to penetrate cancer cells and the high stability, allowing the controlled release of loaded drugs, and protecting drugs from chemical degradation (Coelho et al., 2010).

In the context of OSCC, Bharadwaj et al. (2019) used solid lipid NPs (SLN) loaded with paclitaxel (PTX), 5-FU and ascorbic acid individually to treat OSCC cells. They reported that the combination of SLN loaded with PTX and ascorbic acid respectively exhibited a better therapeutic efficacy in the treatment of OSCC in 4-NQO induced OSCC mouse model. After 2 weeks treatment, pathological analysis showed that dysplastic degree of tumor was decreased. Their results provided valuable insights for the design of novel combinational therapeutic strategies for the treatment of OSCC. Gusti-Ngurah-Putu et al. (2019) evaluated the therapeutic efficacy of PDT + lipid-platinum-chloride nanoparticles (LPC NPs) on a xenograft model of OSCC. Mice treated with PDT + LPC exhibited an significantly reduced tumor volume by up to ∼112% as compared with the control mice, histological analysis confirmed that proliferation index was decreased, but apoptosis rate increased in OSCC cells. In addition, side effects on renal damage was reduced (Gusti-Ngurah-Putu et al., 2019). Their results indicated that combined PDT with LPC NPs could significantly enhance the medicinal outcome in human OSCC.

## 3 Cell membrane-based NPs

Recently, studies revealed that cell membrane-based NPs are an emerging technique that uses cell membranes to directly coat the outermost layer of NPs (Pereira-Silva et al., 2020) and can maximize drug targeting by exploiting the inherited bio-functionalities of cell membranes (CM) extracted from source cells and might potentially enhance the effect of anticancer drugs (Pereira-Silva et al., 2020). Thus, CM-based NPs have received widespread attention in the field of cancer (Sushnitha et al., 2020).

Using this approach, Shi et al. (2020) prepared poly (*β*-amino ester)/PLGA nanoparticles co-loaded with indocyanine green and Nrf2-siRNA and then encapsulated them in vesicles derived from OSCC CM to form M@PPI-siRNA. They reported that photosensitizer indocyanine green (ICG) and Nrf2-siRNA encapsulated within the vesicles of cancer cell CM showed an synergistic anticancer effect of PTT and amplified PDT in oral tongue squamous cell carcinoma cells through an inhibitory effect on the proliferation and stimulatory effect on the apoptosis of oral tongue squamous cell carcinoma cells. Dai et al. (2022) developed the biomimetic nanomaterial PCN-CQ@CCM that could homologously adhere to cancer cells, enhancing the retention and uptake of nanomaterials in the tumor microenvironment. Their results showed that such biomimetic nanomaterial system could synergically potent the effect of PDT by inhibiting macrophage phagocytosis in oral cancer (Dai et al., 2022). Chen et al. (2023b) further synthetized an biomimetic nanomaterial system named cobalt-ferrocene metal–organic framework (Co-Fc) and loaded with the classical autophagy inhibitor hydroxychloroquine (HCQ) (Co-Fc@HCQ) nanoparticles and then constructed with CMM extracted from CAL-27 OSCC cells, which reduced immune escape and macrophage phagocytosis in OSCC cells. These findings indicate that CM may be used as an efficient synergist of PDT for OSCC treatment.

**FIGURE 1:**
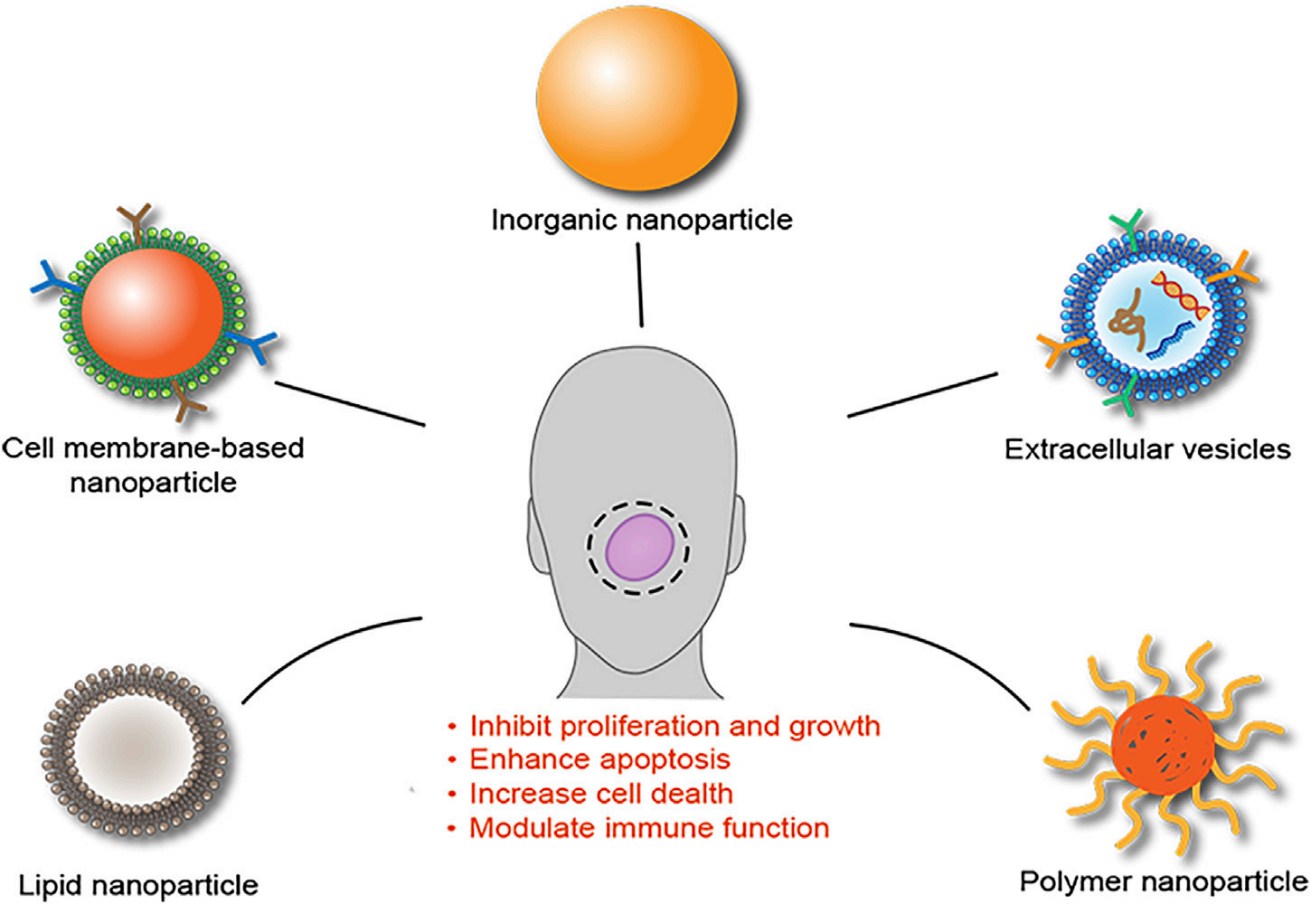
Schematic summary of NPs as DDSs for the treatment of OSCC. Analysis of current studies suggested that several NPs, including inorganic NPs, extracellular vesicles, polymer NPs, lipid NPs, and cell membrane-based NPs, have been developed as DDSs for the treatment of OSCC and showed an enhanced therapeutic efficacy through multiple mechanisms.

Taken together above reviewing data, we have summarized current findings in Figure 1.

## 4 Current challenges of the strategies of NPs as DDSs in OSCC

Despite encouraging findings demonstrating their exciting potential of NPs as DDSs in the treatment of OSCC, however a number of challenges remain. For example, experimental models used *in vivo* and *in vitro* studies were different to human OSCC in the oral environment. Most of *in vitro* models used OSCC cell lines that derived from human OSCC and cultured in experimental media and treated with NPs combined with anticancer drugs, which is much simple than physiological oral condition. Even *in vivo* animal models, whether subcutaneous xenotransplanted mice or *in situ* oral tumor mice, face problems such as lack of normal-precancerous-cancerous process, which is different to the process of human OSCC (Montenegro et al., 2016). Thus, how to develop an experimental model that well mimics the human oral environment and can be used for the verification of *in vivo* animal and *in vitro* cellular findings become necessary. Various novel NPs-based DDSs have been extensively studied. However, *in vivo* bioavailability data was still waiting to be investigated (Sachdeva et al., 2022). Furthermore, studies on long-term toxicity and side effects of NPs in human body are needed to be conducted. Therefore, more detailed studies are required to evaluate biosafety of all types of NPs. Particularly, there is a lack of ongoing and completed clinical trials in patients with OSCC so far (De Felice et al., 2019), which is a necessary step for the definition of the potential oncology applications of NPs as DDSs in daily clinical practice in the treatment of OSCC in the future. Therefore, how to develop NPs as DDSs in a cheaper, easier, and faster way still remains a technical hurdle. Until these issues are resolved, clinical trials can be considered.

Unlike other human cancers, such as gastric, colorectal and lung cancers, OSCC is not rich in angiogenesis, which means that the level of chemotherapeutic drugs (such as paclitaxel and platinum-based drugs) that normally enter the circulation and reach the tumor site, by oral or intravenous administration, is limited (Bhat et al., 2021; Kitamura et al., 2021). In addition, the use of these free drugs is faced with poor therapeutic efficacy and severe side effects. Therefore, NPs-based therapies become particularly suitable for enhancing the bioavailability of chemotherapeutic drugs and target delivery to the tumor site, which can significantly improve the therapeutic efficacy and reduce side effects (De Felice et al., 2019). In addition, unlike tumors located deeply in the human body, such as colon cancer and liver cancer, OSCC is located at surface such as the buccal mucosa and tongue, which makes that OSCC is suitable for combinational treatment with NPs-mediated/enhanced PDT/PTT (Fan et al., 2020b). Also, hydrogels that can be applied to the surface of the skin or placed inside the surgical wound are options for the treatment of OSCC (Sepantafar et al., 2017).

## 5 Conclusion marks and perspectives

OSCC patients with advanced stage are often unresectable and metastatic, therapeutic approaches are commonly chemotherapy, radiotherapy, and biotherapy, which have various limitations and shortcomings and result in a decreased therapeutic response in clinical practice. For example, these conventional therapeutic approaches lack optimal anticancer effects, however, they may induce strong site effects e.g., non-specific cell toxicity that can significantly damage health cells during the treatment period and some patients have to cease therapies. Therefore, the improvement of these therapeutic efficacy become critically important. It is becoming evident that NPs can be used as DDSs and combined with different therapies to achieve an better therapeutic effect in patients with OSCC due to their ultra-small size, functional modification, and responsiveness to heat, light and other conditions as compared with conventional drug delivery approaches. Further work that verifies and confirms the improvement of clinical efficacy of different NPs as DDSs combining with anticancer drugs in treating patients with OSCC is necessary and important.

## Figures and Tables

**Table:** 

**ATRA**	all-trans retinoic acid
**AuNPs or GNPs**	gold nanoparticles
**BMEV**	bitter melon-derived extracellular vesicles
**Cat**	catechol
**Cat-NPs**	catechol (Cat)-modified chitosan/hyaluronic acid (HA) nanoparticles
**CTSB**	capivasertib-encapsulated cathepsin B
**CDDP**	a nanodrug complex containing cetuximab (CTX) and cisplatin
**CDDP-PLA NPs**	PLA combined with CDDP nanoparticles
**CM**	cell memberane
**Co-Fc**	cobalt-ferrocene metal–organic framework
**CRDA**	Cu2−xS-RB@DMSN-AE105
**CQ**	chloroquine
**CTX**	cetuximab
**DDS**	drug delivery system
**DMSN**	dendritic mesoporous silica nanoparticles
**DOX**	doxorubicin
**EGFR**	epithelial growth factor receptor
**EPR**	enhanced permeability and retention
**EXO**	exosomes
**FA**	folic acid
**FA-NPs**	folic acid (FA)-targeted paclitaxel-loaded nanoparticles
**FGFR1i**	fibroblast growth factor receptor1-inhibitor
**GA-AuNPs**	gum arabic encapsulated gold nanoparticles
**GNSbs**	gold nano-sesame beads
**GQDs**	graphene quantum dots
**GSH**	glutathione
**HA**	hyaluronic acid
**HIF-1α**	hypoxia inducible factor-1 *α*
**H-MnO2**	hollow mesoporous MnO2
**ISG**	*in situ* gel
**LPC NPs**	lipid-platinum chloride nanoparticles
**MDR**	multidrug resistance
**MMP-9**	matrix metallopeptidase 9
**MSC**	mesenchymal stromal cell
**MSC-EXO**	mesenchymal stromal cell-derived exosomes
**MSN**	mesoporous silica nanoparticles
**nano-SHAP**	hydroxyapatite nanoparticles
**NF-κB**	nuclear factor-κB
**NIR**	near-infrared
**NPs**	nanoparticles
**NTP**	non-thermal plasma
**OSCC**	oral squamous cell carcinoma
**PCA**	polycarbonate
**PD-L1**	programmed cell death protein ligand 1
**PDT**	photodynamic therapy
**PEG**	polyethylene glycol
**PhCsNPs**	phloretin-loaded chitosan nanoparticles
**PLA**	polylactic acid
**PLGA**	Poly (d,l-lactide-co-glycolide)
**PtNCP**	platinum nanocomposite
**PTT**	photonic hyperthermal therapy
**PTX**	paclitaxel
**RENP**	rare earth nanoparticle
**ROS**	reactive oxygen species
**S-CD**	sulfur-doped carbon dots
**SDT**	sonodynamic therapy
**SIM**	simvastatin
**SPION**	superparamagnetic iron oxide nanoparticles
**TSCC**	tongue squamous cell carcinoma
**UCN**	upconversion nanoparticles
**ZA**	zoledronic acid
**γδ TDE**	γδ T cell-derived extracellular vesicles
**5-FU**	5-fluorouracil

## References

[B1] Abdel Hamid H. M. Darwish Z. E. Elsheikh S. M. Mourad G. M. Donia H. M. Afifi M. M. (2021). Following cytotoxic nanoconjugates from injection to halting the cell cycle machinery and its therapeutic implications in oral cancer. BMC Cancer 21 (1), 170. 10.1186/s12885-021-07849-x 33596850 PMC7890963

[B2] Abed A. Derakhshan M. Karimi M. Shirazinia M. Mahjoubin-Tehran M. Homayonfal M. (2022). Platinum nanoparticles in biomedicine: Preparation, anti-cancer activity, and drug delivery vehicles. Front. Pharmacol. 13, 797804. 10.3389/fphar.2022.797804 35281900 PMC8904935

[B3] Afrasiabi M. Seydi E. Rahimi S. Tahmasebi G. Jahanbani J. Pourahmad J. (2021). The selective toxicity of superparamagnetic iron oxide nanoparticles (SPIONs) on oral squamous cell carcinoma (OSCC) by targeting their mitochondria. J. Biochem. Mol. Toxicol. 35 (6), 1–8. 10.1002/jbt.22769 33704875

[B4] Arias L. S. Pessan J. P. Vieira A. P. M. Lima T. M. T. Delbem A. C. B. Monteiro D. R. (2018). Iron oxide nanoparticles for biomedical applications: A perspective on synthesis, drugs, antimicrobial activity, and toxicity. Antibiotics 7 (2), 46. 10.3390/antibiotics7020046 29890753 PMC6023022

[B5] Batrakova E. V. Kim M. S. (2015). Using exosomes, naturally-equipped nanocarriers, for drug delivery. J. Control Release 219, 396–405. 10.1016/j.jconrel.2015.07.030 26241750 PMC4656109

[B6] Bharadwaj R. Sahu B. P. Haloi J. Laloo D. Barooah P. Keppen C. (2019). Combinatorial therapeutic approach for treatment of oral squamous cell carcinoma. Artif. Cells Nanomed Biotechnol. 47 (1), 572–585. 10.1080/21691401.2019.1573176 30831033

[B7] Bhat A. A. Yousuf P. Wani N. A. Rizwan A. Chauhan S. S. Siddiqi M. A. (2021). Tumor microenvironment: An evil nexus promoting aggressive head and neck squamous cell carcinoma and avenue for targeted therapy. Signal Transduct. Target. Ther. 6 (1), 12. 10.1038/s41392-020-00419-w 33436555 PMC7804459

[B8] Bugshan A. Farooq I. (2020). Oral squamous cell carcinoma: Metastasis, potentially associated malignant disorders, etiology and recent advancements in diagnosis. F1000Res. 9, 229. 10.12688/f1000research.22941.1 32399208 PMC7194458

[B9] Calixto G. Bernegossi J. Fonseca-Santos B. Chorilli M. (2014). Nanotechnology-based drug delivery systems for treatment of oral cancer: A review. Int. J. Nanomedicine 9, 3719–3735. 10.2147/IJN.S61670 25143724 PMC4134022

[B10] Chakraborty S. Suresh T. N. R. Mohiyuddin A. S. (2023). Role of matrix metalloproteinase 9 in predicting lymph node metastases in oral squamous cell carcinoma. Cureus 15 (1), e33495. 10.7759/cureus.33495 36756017 PMC9902810

[B11] Charbe N. B. Amnerkar N. D. Ramesh B. Tambuwala M. M. Bakshi H. A. Aljabali A. A. A. (2020). Small interfering RNA for cancer treatment: Overcoming hurdles in delivery. Acta Pharm. Sin. B 10 (11), 2075–2109. 10.1016/j.apsb.2020.10.005 33304780 PMC7714980

[B12] Chen J. Zhu Z. Pan Q. Bai Y. Yu M. Zhou Y. (2023). Targeted therapy of oral squamous cell carcinoma with cancer cell membrane coated Co-fc nanoparticles via autophagy inhibition. Adv. Funct. Mater. 2023, 2300235. 10.1002/adfm.202300235

[B13] Chen L. Kong Q. Tian M. Zhang Q. Xia C. Deng C. (2023). Zn(0.4)Mg(0.6)Fe(2)O(4) nanoenzyme: A novel chemo-sensitizer for the chemotherapy treatment of oral squamous cell carcinoma. Nanoscale Adv. 5 (3), 851–860. 10.1039/d2na00750a 36756528 PMC9890649

[B14] Chen M. H. Chen M. H. Li C. Y. Tung F. I. Chen S. Y. Liu T. Y. (2021). Using gold-nanorod-filled mesoporous silica nanobeads for enhanced radiotherapy of oral squamous carcinoma. Nanomaterials 11 (9), 2235. 10.3390/nano11092235 34578551 PMC8472528

[B15] Chen X. J. Zhang X. Q. Tang M. X. Liu Q. Zhou G. (2020). Anti-PD-L1-modified and ATRA-loaded nanoparticles for immuno-treatment of oral dysplasia and oral squamous cell carcinoma. Nanomedicine (Lond) 15 (10), 951–968. 10.2217/nnm-2019-0397 32255397

[B16] Coelho J. F. Ferreira P. C. Alves P. Cordeiro R. Fonseca A. C. Gois J. R. (2010). Drug delivery systems: Advanced technologies potentially applicable in personalized treatments. EPMA J. 1 (1), 164–209. 10.1007/s13167-010-0001-x 23199049 PMC3405312

[B17] Dai H. Yan H. Dong F. Zhang L. Du N. Sun L. (2022). Tumor-targeted biomimetic nanoplatform precisely integrates photodynamic therapy and autophagy inhibition for collaborative treatment of oral cancer. Biomater. Sci. 10 (6), 1456–1469. 10.1039/d1bm01780b 35048086

[B18] Das R. K. Panda S. Bhol C. S. Bhutia S. K. Mohapatra S. (2019). N-doped carbon quantum dot (NCQD)-Deposited carbon capsules for synergistic fluorescence imaging and photothermal therapy of oral cancer. Langmuir 35 (47), 15320–15329. 10.1021/acs.langmuir.9b03001 31682135

[B19] De Felice F. Cavallini C. Barlattani A. Tombolini M. Brugnoletti O. Tombolini V. (2019). Nanotechnology in oral cavity carcinoma: Recent trends and treatment opportunities. Nanomater. (Basel) 9 (11), 1546. 10.3390/nano9111546 PMC6915589 31683582

[B20] Deshmukh V. Shekar K. (2021). “Oral squamous cell carcinoma: Diagnosis and treatment planning,” in Oral and maxillofacial surgery for the clinician. Editors Bonanthaya K. Panneerselvam E. Manuel S. Kumar V. V. Rai A. (Singapore: Springer Nature Singapore), 1853–1867.

[B21] Elsharkasy O. M. Nordin J. Z. Hagey D. W. de Jong O. G. Schiffelers R. M. Andaloussi S. E. (2020). Extracellular vesicles as drug delivery systems: Why and how?. Adv. Drug Deliv. Rev. 159, 332–343. 10.1016/j.addr.2020.04.004 32305351

[B22] Enășescu D. A. Moisescu M. G. Imre M. Greabu M. Ripszky Totan A. Stanescu-Spinu I. (2021). Lutein treatment effects on the redox status and metalloproteinase-9 (MMP-9) in oral cancer squamous cells—are there therapeutical hopes?. Materials 14 (11), 2968. 10.3390/ma14112968 34072756 PMC8199462

[B23] Endo K. Ueno T. Kondo S. Wakisaka N. Murono S. Ito M. (2013). Tumor-targeted chemotherapy with the nanopolymer-based drug NC-6004 for oral squamous cell carcinoma. Cancer Sci. 104 (3), 369–374. 10.1111/cas.12079 23216802 PMC7657215

[B24] Essawy M. M. El-Sheikh S. M. Raslan H. S. Ramadan H. S. Kang B. Talaat I. M. (2021). Function of gold nanoparticles in oral cancer beyond drug delivery: Implications in cell apoptosis. Oral Dis. 27 (2), 251–265. 10.1111/odi.13551 32657515

[B25] Fan H. Y. Zhu Z. L. Zhang W. L. Yin Y. J. Tang Y. L. Liang X. H. (2020). Light stimulus responsive nanomedicine in the treatment of oral squamous cell carcinoma. Eur. J. Med. Chem. 199, 112394. 10.1016/j.ejmech.2020.112394 32402938

[B26] Fan L. Wang J. Xia C. Zhang Q. Pu Y. Chen L. (2020). Glutathione-sensitive and folate-targeted nanoparticles loaded with paclitaxel to enhance oral squamous cell carcinoma therapy. J. Mater Chem. B 8 (15), 3113–3122. 10.1039/c9tb02818h 32207763

[B27] Gamal-Eldeen A. M. Baghdadi H. M. Afifi N. S. Ismail E. M. Alsanie W. F. Althobaiti F. (2021). Gum Arabic-encapsulated gold nanoparticles modulate hypoxamiRs expression in tongue squamous cell carcinoma. Mol. Cell. Toxicol. 17 (2), 111–121. 10.1007/s13273-021-00117-w

[B28] Gulland A. (2016). Oral cancer rates rise by two thirds. BMJ 355, i6369. 10.1136/bmj.i6369 27887000

[B29] Gusti-Ngurah-Putu E. P. Huang L. Hsu Y. C. (2019). Effective combined photodynamic therapy with lipid platinum chloride nanoparticles therapies of oral squamous carcinoma tumor inhibition. J. Clin. Med. 8 (12), 2112. 10.3390/jcm8122112 31810241 PMC6947167

[B30] Ketabat F. Pundir M. Mohabatpour F. Lobanova L. Koutsopoulos S. Hadjiiski L. (2019). Controlled drug delivery systems for oral cancer treatment-current status and future perspectives. Pharmaceutics 11 (7), 302. 10.3390/pharmaceutics11070302 31262096 PMC6680655

[B31] Kitamura N. Sento S. Yoshizawa Y. Sasabe E. Kudo Y. Yamamoto T. (2021). Current trends and future prospects of molecular targeted therapy in head and neck squamous cell carcinoma. Int. J. Mol. Sci. 22 (1), 240. 10.3390/ijms22010240 PMC7795499 33383632

[B32] Kurakula M. Naveen N. R. (2020). *In situ* gel loaded with chitosan-coated simvastatin nanoparticles: Promising delivery for effective anti-proliferative activity against tongue carcinoma. Mar. Drugs 18 (4), 201. 10.3390/md18040201 32283782 PMC7231276

[B33] Lang L. Lam T. Chen A. Jensen C. Duncan L. Kong F. C. (2020). Circumventing AKT-associated radioresistance in oral cancer by novel nanoparticle-encapsulated capivasertib. Cells 9 (3), 533. 10.3390/cells9030533 32106632 PMC7140405

[B34] Li L. Lu S. Liang X. Cao B. Wang S. Jiang J. (2019). γδTDEs: An efficient delivery system for miR-138 with anti-tumoral and immunostimulatory roles on oral squamous cell carcinoma. Mol. Ther. Nucleic Acids 14, 101–113. 10.1016/j.omtn.2018.11.009 30594069 PMC6307324

[B35] Li Q. Liu X. Yan W. Chen Y. (2020). Antitumor effect of poly lactic acid nanoparticles loaded with cisplatin and chloroquine on the oral squamous cell carcinoma. Aging (Albany NY) 13 (2), 2593–2603. 10.18632/aging.202297 33323546 PMC7880364

[B36] Li Q. Zhou R. Xie Y. Li Y. Chen Y. Cai X. (2020). Sulphur-doped carbon dots as a highly efficient nano-photodynamic agent against oral squamous cell carcinoma. Cell Prolif. 53 (4), e12786. 10.1111/cpr.12786 32301195 PMC7162798

[B37] Lin B. Wu J. Wang Y. Sun S. Yuan Y. Tao X. (2021). Peptide functionalized upconversion/NIR II luminescent nanoparticles for targeted imaging and therapy of oral squamous cell carcinoma. Biomater. Sci. 9 (3), 1000–1007. 10.1039/d0bm01737j 33305773

[B38] Liu Z. Shi J. Zhu B. Xu Q. (2020). Development of a multifunctional gold nanoplatform for combined chemo-photothermal therapy against oral cancer. Nanomedicine (Lond) 15 (7), 661–676. 10.2217/nnm-2019-0415 32141806

[B39] Lucky S. S. Idris N. M. Huang K. Kim J. Li Z. Thong P. S. (2016). *In vivo* biocompatibility, biodistribution and therapeutic efficiency of titania coated upconversion nanoparticles for photodynamic therapy of solid oral cancers. Theranostics 6 (11), 1844–1865. 10.7150/thno.15088 27570555 PMC4997241

[B40] Mabrouk A. A. El-Mezayen N. S. Tadros M. I. El-Gazayerly O. N. El-Refaie W. M. (2023). Novel mucoadhesive celecoxib-loaded cubosomal sponges: Anticancer potential and regulation of myeloid-derived suppressor cells in oral squamous cell carcinoma. Eur. J. Pharm. Biopharm. 182, 62–80. 10.1016/j.ejpb.2022.12.003 36513316

[B41] Mariadoss A. V. A. Vinayagam R. Senthilkumar V. Paulpandi M. Murugan K. Xu B. (2019). Phloretin loaded chitosan nanoparticles augments the pH-dependent mitochondrial-mediated intrinsic apoptosis in human oral cancer cells. Int. J. Biol. Macromol. 130, 997–1008. 10.1016/j.ijbiomac.2019.03.031 30844461

[B42] Montenegro M. F. Sundqvist M. L. Nihlen C. Hezel M. Carlstrom M. Weitzberg E. (2016). Profound differences between humans and rodents in the ability to concentrate salivary nitrate: Implications for translational research. Redox Biol. 10, 206–210. 10.1016/j.redox.2016.10.011 27810735 PMC5094378

[B43] Mukherjee D. Dash P. Ramadass B. Mangaraj M. (2022). Nanocurcumin in oral squamous cancer cells and its efficacy as a chemo-adjuvant. Cureus 14 (5), e24678. 10.7759/cureus.24678 35663647 PMC9162890

[B44] Murata T. Kutsuna T. Kurohara K. Shimizu K. Tomeoku A. Arai N. (2018). Evaluation of a new hydroxyapatite nanoparticle as a drug delivery system to oral squamous cell carcinoma cells. Anticancer Res. 38 (12), 6715–6720. 10.21873/anticanres.13040 30504381

[B45] Nakamura Y. Mochida A. Choyke P. L. Kobayashi H. (2016). Nanodrug delivery: Is the enhanced permeability and retention effect sufficient for curing cancer?. Bioconjug Chem. 27 (10), 2225–2238. 10.1021/acs.bioconjchem.6b00437 27547843 PMC7397928

[B46] Nandini D. B. Rao R. S. Hosmani J. Khan S. Patil S. Awan K. H. (2020). Novel therapies in the management of oral cancer: An update. Dis. Mon. 66 (12), 101036. 10.1016/j.disamonth.2020.101036 32594997

[B47] Ortega A. da Silva A. B. da Costa L. M. Zatta K. C. Onzi G. R. da Fonseca F. N. (2023). Thermosensitive and mucoadhesive hydrogel containing curcumin-loaded lipid-core nanocapsules coated with chitosan for the treatment of oral squamous cell carcinoma. Drug Deliv. Transl. Res. 13 (2), 642–657. 10.1007/s13346-022-01227-1 36008703

[B48] Park J. Jang Y.-S. Choi J.-H. Ryu M. Kim G.-C. Byun J. H. (2021). Anticancer efficacy of nonthermal plasma therapy combined with PD-L1 antibody conjugated gold nanoparticles on oral squamous cell carcinoma. Appl. Sci. 11 (10), 4559. 10.3390/app11104559

[B49] Pereira-Silva M. Santos A. C. Conde J. Hoskins C. Concheiro A. Alvarez-Lorenzo C. (2020). Biomimetic cancer cell membrane-coated nanosystems as next-generation cancer therapies. Expert Opin. Drug Deliv. 17 (11), 1515–1518. 10.1080/17425247.2020.1813109 32812476

[B50] Pornpitchanarong C. Rojanarata T. Opanasopit P. Ngawhirunpat T. Patrojanasophon P. (2020). Catechol-modified chitosan/hyaluronic acid nanoparticles as a new avenue for local delivery of doxorubicin to oral cancer cells. Colloids Surf. B Biointerfaces 196, 111279. 10.1016/j.colsurfb.2020.111279 32750605

[B51] Qiu Y. Sun J. Qiu J. Chen G. Wang X. Mu Y. (2020). Antitumor activity of cabazitaxel and MSC-TRAIL derived extracellular vesicles in drug-resistant oral squamous cell carcinoma. Cancer Manag. Res. 12, 10809–10820. 10.2147/CMAR.S277324 33149686 PMC7605918

[B52] Ran J. Liu T. Song C. Wei Z. Tang C. Cao Z. (2023). Rhythm mild-temperature photothermal therapy enhancing immunogenic cell death response in oral squamous cell carcinoma. Adv. Healthc. Mater 12 (6), e2202360. 10.1002/adhm.202202360 36401600

[B53] Rizvi S. A. A. Saleh A. M. (2018). Applications of nanoparticle systems in drug delivery technology. Saudi Pharm. J. 26 (1), 64–70. 10.1016/j.jsps.2017.10.012 29379334 PMC5783816

[B54] Sachdeva A. Dhawan D. Jain G. K. Yerer M. B. Collignon T. E. Tewari D. (2022). Novel strategies for the bioavailability augmentation and efficacy improvement of natural products in oral cancer. Cancers (Basel) 15 (1), 268. 10.3390/cancers15010268 36612264 PMC9818473

[B55] Sah A. K. Vyas A. Suresh P. K. Gidwani B. (2018). Application of nanocarrier-based drug delivery system in treatment of oral cancer. Artif. Cells Nanomed Biotechnol. 46 (4), 650–657. 10.1080/21691401.2017.1373284 28880679

[B56] Sepantafar M. Maheronnaghsh R. Mohammadi H. Radmanesh F. Hasani-sadrabadi M. M. Ebrahimi M. (2017). Engineered hydrogels in cancer therapy and diagnosis. Trends Biotechnol. 35 (11), 1074–1087. 10.1016/j.tibtech.2017.06.015 28734545

[B57] Shi S. Wang Y. Wang B. Chen Q. Wan G. Yang X. (2020). Homologous-targeting biomimetic nanoparticles for photothermal therapy and Nrf2-siRNA amplified photodynamic therapy against oral tongue squamous cell carcinoma. Chem. Eng. J. 388, 124268. 10.1016/j.cej.2020.124268

[B58] Shi X. L. Li Y. Zhao L. M. Su L. W. Ding G. (2019). Delivery of MTH1 inhibitor (TH287) and MDR1 siRNA via hyaluronic acid-based mesoporous silica nanoparticles for oral cancers treatment. Colloids Surf. B Biointerfaces 173, 599–606. 10.1016/j.colsurfb.2018.09.076 30352381

[B59] Siegel R. L. Miller K. D. Jemal A. (2020). Cancer statistics, 2020. CA Cancer J. Clin. 70 (1), 7–30. 10.3322/caac.21590 31912902

[B60] Singh P. Pandit S. Mokkapati V. R. S. S. Garg A. Ravikumar V. Mijakovic I. (2018). Gold nanoparticles in diagnostics and therapeutics for human cancer. Int. J. Mol. Sci. 19 (7), 1979. 10.3390/ijms19071979 29986450 PMC6073740

[B61] Srivastava S. Gupta S. Mohammad S. Ahmad I. (2019). Development of alpha-tocopherol surface-modified targeted delivery of 5-fluorouracil-loaded poly-D, L-lactic-co-glycolic acid nanoparticles against oral squamous cell carcinoma. J. Cancer Res. Ther. 15 (3), 480–490. 10.4103/jcrt.JCRT_263_18 31169208

[B62] Sung H. Ferlay J. Siegel R. L. Laversanne M. Soerjomataram I. Jemal A. (2021). Global cancer statistics 2020: GLOBOCAN estimates of incidence and mortality worldwide for 36 cancers in 185 countries. CA Cancer J. Clin. 71 (3), 209–249. 10.3322/caac.21660 33538338

[B63] Surer S. I. Elcitepe T. B. Akcay D. Daskin E. Calibasi Kocal G. Arican Alicikus Z. (2021). A promising, novel radiosensitizer nanodrug complex for oral cavity cancer: Cetuximab and cisplatin-conjugated gold nanoparticles. Balk. Med. J. 38 (5), 278–286. 10.5152/balkanmedj.2021.21013 PMC8880916 34462254

[B64] Sushnitha M. Evangelopoulos M. Tasciotti E. Taraballi F. (2020). Cell membrane-based biomimetic nanoparticles and the immune system: Immunomodulatory interactions to therapeutic applications. Front. Bioeng. Biotechnol. 8, 627. 10.3389/fbioe.2020.00627 32626700 PMC7311577

[B65] Tanaka M. Okinaga T. Iwanaga K. Matsuo K. Toyono T. Sasaguri M. (2019). Anticancer effect of novel platinum nanocomposite beads on oral squamous cell carcinoma cells. J. Biomed. Mater Res. B Appl. Biomater. 107 (7), 2281–2287. 10.1002/jbm.b.34320 30689290

[B66] Wang F. Li C. Cheng J. Yuan Z. (2016). Recent advances on inorganic nanoparticle-based cancer therapeutic agents. Int. J. Environ. Res. Public Health 13 (12), 1182. 10.3390/ijerph13121182 27898016 PMC5201323

[B67] Wang Y. Zhao Q. Han N. Bai L. Li J. Liu J. (2015). Mesoporous silica nanoparticles in drug delivery and biomedical applications. Nanomedicine 11 (2), 313–327. 10.1016/j.nano.2014.09.014 25461284

[B68] Wiklander O. P. Nordin J. Z. O'Loughlin A. Gustafsson Y. Corso G. Mager I. (2015). Extracellular vesicle *in vivo* biodistribution is determined by cell source, route of administration and targeting. J. Extracell. Vesicles 4, 26316. 10.3402/jev.v4.26316 25899407 PMC4405624

[B69] Xia W. Tao Z. Zhu B. Zhang W. Liu C. Chen S. (2021). Targeted delivery of drugs and genes using polymer nanocarriers for cancer therapy. Int. J. Mol. Sci. 22 (17), 9118. 10.3390/ijms22179118 34502028 PMC8431379

[B70] Yang M. Luo Q. Chen X. Chen F. (2021). Bitter melon derived extracellular vesicles enhance the therapeutic effects and reduce the drug resistance of 5-fluorouracil on oral squamous cell carcinoma. J. Nanobiotechnology 19 (1), 259. 10.1186/s12951-021-00995-1 34454534 PMC8400897

[B71] Yu A. J. Choi J. S. Swanson M. S. Kokot N. C. Brown T. N. Yan G. (2019). Association of race/ethnicity, stage, and survival in oral cavity squamous cell carcinoma: A seer study. OTO Open 3 (4), 2473974X19891126. 10.1177/2473974X19891126 PMC6904786 31840132

[B72] Yunxia L. Wenjuan D. Zhiguang F. Huanhuan W. Jiexin W. Yuan L. (2018). pH-Responsive polycarbonate copolymer-based nanoparticles for targeted anticancer drug delivery. Chem. Res. Chin. Univ. 34, 1041–1050. 10.1007/s40242-018-8147-5

[B73] Zhang M. Liang J. Yang Y. Liang H. Jia H. Li D. (2020). Current trends of targeted drug delivery for oral cancer therapy. Front. Bioeng. Biotechnol. 8, 618931. 10.3389/fbioe.2020.618931 33425881 PMC7793972

[B74] Zhang Q. Xiao Q. Yin H. Xia X. Pu Y. He Z. (2020). Milk-exosome based pH/light sensitive drug system to enhance anticancer activity against oral squamous cell carcinoma. RSC Adv. 1 (47), 28314–28323. 10.1039/d0ra05630h PMC9055635 35519132

[B75] Zhang X. Li H. Yi C. Chen G. Li Y. Zhou Y. (2020). Host immune response triggered by graphene quantum-dot-mediated photodynamic therapy for oral squamous cell carcinoma. Int. J. Nanomedicine 15, 9627–9638. 10.2147/IJN.S276153 33293811 PMC7718975

[B76] Zhou Z.-H. Liang S.-Y. Zhao T.-C. Chen X.-Z. Cao X.-K. Qi M. (2021). Overcoming chemotherapy resistance using pH-sensitive hollow MnO2 nanoshells that target the hypoxic tumor microenvironment of metastasized oral squamous cell carcinoma. J. Nanobiotechnology 19 (1), 157. 10.1186/s12951-021-00901-9 34039370 PMC8157461

[B77] Zuo J. Huo M. Wang L. Li J. Chen Y. Xiong P. (2020). Photonic hyperthermal and sonodynamic nanotherapy targeting oral squamous cell carcinoma. J. Mater Chem. B 8, 9084–9093. 10.1039/d0tb01089h 32926057

